# The Connection between Gut and Lung Microbiota, Mast Cells, Platelets and SARS-CoV-2 in the Elderly Patient

**DOI:** 10.3390/ijms232314898

**Published:** 2022-11-28

**Authors:** Giovanna Traina

**Affiliations:** Department of Pharmaceutical Sciences, University of Perugia, Via Romana, 06126 Perugia, Italy; giovanna.traina@unipg.it

**Keywords:** SARS-CoV-2, inflammation, intestinal microbiota, immune protection, mast cells, platelets, aging

## Abstract

The human coronavirus SARS-CoV-2 or COVID-19 that emerged in late 2019 causes a respiratory tract infection and has currently resulted in more than 627 million confirmed cases and over 6.58 million deaths worldwide up to October 2022. The highest death rate caused by COVID-19 is in older people, especially those with comorbidities. This evidence presents a challenge for biomedical research on aging and also identifies some key players in inflammation, including mast cells and platelets, which could represent important markers and, at the same time, unconventional therapeutic targets. Studies have shown a decrease in the diversity of gut microbiota composition in the elderly, particularly a reduced abundance of butyrate-producing species, and COVID-19 patients manifest faecal microbiome alterations, with an increase in opportunistic pathogens and a depletion of commensal beneficial microorganisms. The main purpose of this narrative review is to highlight how an altered condition of the gut microbiota, especially in the elderly, could be an important factor and have a strong impact in the lung homeostasis and COVID-19 phenomenon, jointly to the activation of mast cells and platelets, and also affect the outcomes of the pathology. Therefore, a targeted and careful control of the intestinal microbiota could represent a complementary intervention to be implemented for the management and the challenge against COVID-19.

## 1. Introduction

The human coronavirus SARS-CoV-2 or COVID-19 that emerged in late 2019 causes a respiratory tract infection of the COVID-19 disease and, according to the official website of the Ministry of Health, which reports World Health Organization data, has currently resulted in more than 627 million confirmed cases and over 6.58 million deaths worldwide up to October 2022.

The characteristic symptoms presented by patients affected by SARS-CoV-2 led to the belief that it was a pneumonia with an interstitial component, very often bilateral, associated with respiratory symptoms which in the early phase are generally limited, but which can subsequently lead to progressive clinical instability with respiratory failure. The phenomenon of the so-called “silent hypoxemia”, characterized by low blood oxygenation values in the absence of subjective feeling of dyspnea, is characteristic of this phase of the disease. This scenario, in a number of people, can evolve towards a worsening clinical picture dominated by a cytokine storm, the excessive immune response from the uncontrolled release of a series of interleukins, chemokines, interferons, and tumour necrosis factors and the consequent hyperinflammatory state, which determines local and systemic consequences. Such a response represents a negative prognostic factor producing, at the pulmonary level, pictures of arterial and venous thrombi of small vessels and evolution towards severe and sometimes permanent pulmonary lesions (pulmonary fibrosis) [1]. In particular, vascular permeability is increased, resulting in a large amount of fluid and blood cells entering the alveoli, causing dyspnoea and even respiratory failure, desquamation of alveolar cells and hyaline membrane formation. A mass of fluid similar to mucus accumulates in the lungs, and this accumulation is caused by an excessive immune response due to signalling molecules, in particular interleukin-(IL)-6, IL-8, and tumour necrosis factor (TNFα) [1,2,3,4]. Cytokine overproduction and cytokine storm induce clinically relevant extrapulmonary effects on various key organs such as heart, kidney, liver and intestine and dysbiosis [3]. The final stages of this very severe clinical picture can lead to multi-organ failure, with cardiovascular, gastrointestinal, haematological, respiratory, neurological and renal complications [5].

### 1.1. SARS-CoV-2: An Overview

Coronaviruses (CoVs) belong to the coronaviridae family, which comprises a group of positive-enveloped single-stranded RNA viruses. These viruses have the largest genome among RNA viruses, and morphologically they appear as surrounded by a corona under the electron microscope [6].

Like other coronaviruses, SARS-CoV-2 has four structural proteins, known as: protein S (ear or spinule), E (envelope), M (membrane) and N (nucleocapsid); the N protein contains the RNA genome while the S, E and M proteins together create the viral capsid. Specifically, there are three protein components of the viral envelope. The most important of these is the S-glycoprotein (Spike), a very large transmembrane protein that mediates attachment to the receptor and the fusion of the cell membrane of the host cell with that of the virus. M-glycoprotein is the most abundant constituent of CoVs and shapes the virion envelope. Protein E is a small polypeptide, and due to its small size and limited amount, E was detected much later than other structural proteins [6].

### 1.2. Imbalance of Renin Angiotensin System

The renin angiotensin system (RAS) is a well-known physiological system responsible for controlling cardiovascular dynamics through the modulation of blood pressure. In particular, angiotensinogen is converted into angiotensin I by renin, produced in the kidneys. Angiotensin I is transformed into angiotensin II by an extracellular angiotensin converting enzyme (ACE). Angiotensin II binds to the G protein-coupled receptor (GPCR), angiotensin II type 1 receptor (AT1R) in order to initiate its physiological functions. In general, the activation of AT1R by angiotensin II causes several physiologically important events including vasoconstriction, inflammation, thrombosis and production of reactive oxygen species (ROS). Angiotensin II is further degraded into angiotensin 1–7 by the action of the ACE2 enzyme. Angiotensin 1–7 binds to another GPCR and induces physiological events essentially opposite to those induced by AT1R activation, which include vasodilation, anti-inflammatory, antifibrosis, antithrombosis and ROS neutralization. ACE2 plays a key role as a negative regulator in the overall RAS pathway, exerting protective functions in various RAS-based models of pathogenesis. ACE2 also limits the expression by macrophages of several proinflammatory cytokines.

In the pulmonary phase of COVID-19, SARS-CoV-2 enters the type 2 pneumocyte by inducing the internalization of ACE2 and resulting in down-regulation and deficiency of ACE2. SARS-CoV-2-induced ACE2 deficiency reduces the conversion of angiotensin II to angiotensinogen 1–7 and increases the availability of angiotensin II. Excessive angiotensin II causes AT1R to over-activate, resulting in an imbalance of RAS. However, it should be remembered that SARS-CoV-2 invades host cells via two receptors: ACE2 and through cluster of differentiation 147 (CD147) transmembrane protein mediated endocytosis [7].

Although it has a lower affinity for the COVID-19 virus than ACE2, CD127 specifically accounts for the increase in blood glucose in infected patients, the risk of delayed COVID-19 in women, the increased susceptibility in geriatrics, and the increased susceptibility to T lymphocyte infections [8].

### 1.3. COVID-19 and Inflammation in the Elderly

Inflammation is a complex and multifactorial phenomenon that involves several trigger mechanisms. An inflammatory state underlies a wide variety of diseases, pain, stress, and depression, and an exacerbated inflammatory response may drive the deleterious consequences of the infection [9,10].

Since pre-existing chronic inflammatory conditions such as hypertension, diabetes, obesity, cardiovascular disease, as well as autoimmune diseases also activate the RAS pathway, COVID-19 patients show a significant association between ACE2 deficiency and the clinical severity of these comorbidities. In addition, a decrease in ACE2 expression with age has clinical implications for the poor prognosis of elderly COVID-19 patients. Since the ACE2 gene is located on the X chromosome, the high male mortality rate in COVID-19 patients has been hypothesized to be related to the lower levels of ACE2 gene expression in male patients, so much so that the restoration of SARS-CoV-2-induced RAS imbalance has been suggested as an ideal clinical approach to slow the early progression of COVID-19 pathogenesis [11]. In addition, IL-6, which is one of the cytokines most expressed in the COVID-19 patient, is a multi-effective cytokine with both anti-inflammatory and pro-inflammatory roles. Elevated IL-6 level in COVID-19 patients is a predictor of higher mortality rates [10]. Moreover, IL-6 has been reported to facilitate CD147 expression [12].

In general, proinflammatory cytokines increase the expression of cell adhesion molecules on the surface of neutrophils and endothelial cells. This promotes intercellular interactions. Furthermore, the increased permeability of the pulmonary endothelium and the reduction in barrier protection attracts more neutrophils to the site of infection through endothelial penetration. Such dysregulation of the inflammatory immune response prevents the activation of the adaptive immune response [13,14].

In this perspective, it is very important to characterize the host–pathogen relationship, including immunoprotection correlates, such as COVID-19 virus-specific antibodies that limit disease and correlates of immune dysregulation, such as overproduction of cytokines that can promote disease.

It is known that a patient in old age suffers stressogenic conditions, both those linked to the action of the virus and those attributable to the awareness of the pathology, as well as the physiological conditions linked to the elderly state. Due to an age-altered immune system but also from rather frequent nutritional deficiencies, elderly people are particularly exposed to the risk of infection [15]. In addition, studies reported that visceral fat increases with age, and visceral fat inflammation increases the risk of COVID-19- related complications [16,17].

The semeiology of infections is sometimes atypical in the elderly and the signs and symptoms appear more discreetly than in young adults, leading to diagnostic and therapeutic delay that further aggravates the prognosis of infectious diseases in elderly patients.

Innate immunity serves at the first line of antiviral defence and the largest number of immune cells reside in the intestinal system. However, impaired immune responses in the elderly are responsible for many diseases, as well as increased susceptibility to infections. The response to COVID-19 includes various innate and adaptive traits, such as changes in the composition of dendritic cells and B cells and deeply altered T cell phenotypes that could impair immunoprotective T cells immunity [18]. Recent studies have highlighted the role of adaptive immunity like T cells and B cells in COVID-19. Laing et al. [18] showed reduced T cell immunity in COVID-19 patients. It has been suggested that immunological interventions targeting early predictive inflammatory markers would be more beneficial than those that block the late cytokine-related storm and therefore, very importantly, personalized therapeutic intervention would be required for each patient. Yet, an excessive quantity of neutrophils is associated with the course and severity of COVID-19 [19,20].

Recognition of pathogen-associated molecular patterns (PAMPs) by host pattern recognition receptors (PRRs) is the first step in activating the innate immune system against viral infection. These receptors include, among others, nucleotide-binding oligomerization domain-like receptors (NODs), and toll-like receptors (TLRs). This virus-derived PAMP recognition by innate immune receptors activates a series of signalling cascades that ultimately lead to the activation of transcription factors such as nuclear factor-kappa B (NF-kB) and interferon regulatory factors.

COVID-19 patients show general lymphopenia, which is a significant reduction in the overall number of circulating lymphocytes in the blood [21]. In contrast, the monocyte-macrophage system is significantly upregulated by SARS-CoV-2 infection. In general, monocytes are innate immune cells that participate in inflammatory responses, phagocytosis, antigen presentation, and a variety of other immune processes. Circulating monocytes pour into peripheral tissues to differentiate into macrophages or dendritic cells during inflammation. Thus, the upregulation of monocytes by SARS-CoV-2 infection may contribute to the improvement of proinflammatory processes. Neutrophils are also recruited to the site of infection through the circulation and permeabilization of the endothelial membranes adjacent to the site of infection [1,2,3]. However, due to lymphopenia, COVID-19 patients are more vulnerable to microbial infection. It has been speculated that the inability to eradicate SARS-CoV-2 infection due to its innate immune response antagonism hyper-inflates the innate immune system. This causes an excessive release of inflammatory cytokines to compensate for the depletion of the immune system due to SARS-CoV-2-induced lymphopenia. Finally, the overproduction of cytokines increases the membrane permeability of the capillary walls around the infected alveoli, causing pulmonary oedema, dyspnoea and hypoxemia. The introduction of plasma fluid into the alveoli as well as the loss of elasticity due to the reduced production of surfactant cause complications.

Importantly, SARS-CoV-2 primarily enters through the respiratory tract to infect humans, but it can also enter through the gastrointestinal tract [22].

## 2. Intestinal Microbiota and Systemic Protection

In this context, the involvement of the gut microbiota could play an important role in the COVID-19 phenomenon perhaps not yet sufficiently considered. From a clinical point of view, the elderly patient presents a dysregulation of microbial homeostasis and neurodegeneration that can lead to a condition of greater fragility. This condition manifests itself as a reduced functional reserve, reduced resistance to stress, increased susceptibility to disease, mood, and increased risk of adverse health outcomes [23]. The gut could serve as a reservoir for acute respiratory syndrome COVID-19. Evidence reported that the intestinal microbiota is really altered in SARS-CoV-2 infection [24,25]. In addition, Zuo et al. found that the loss of beneficial species in SARS-CoV-2 persists for a long time in most patients suggesting that exposure to SARS-CoV-2 infection and/or hospitalization may be associated with lasting damage to the intestinal microbiota [25]. In particular, a condition of dysbiosis, perturbations in the structural and, therefore, functional dynamics of the intestinal microbiota, could have a crucial role in COVID-19 disease. Physiologically, the microbiota protects the intestine from colonization of exogenous pathogens and potentially dangerous autochthonous microorganisms. Man has evolved side by side with microbes. The mammalian intestine is colonized by trillions of microorganisms, and most of these are bacteria that evolved together with the host in a symbiotic relationship, ensuring the state of immunosurveillance of the organism. It is well-known that the microbiota can modulate the innate and the adaptive immune system [26,27,28].

Aging implies an imbalanced immunological response to microbial infection associated with elevated levels of several cytokines, including IL-1, IL-6 and TNFα [29,30]. In addition, changes in the expression of PRRs, activation of such receptors by endogenous ligands associated with cellular damage, and unusual downstream signalling events of PRRs activation have evolved to induce a chronic cytokine secretion [31].

The conditions of the intestinal microbiota and pulmonary changes are closely related to immune responses [32]. Interestingly, SARS-CoV-2 leverages the ACE2 receptor to access the host, and this receptor is expressed in both the respiratory and gut tracts [33]. ACE2 is involved in controlling intestinal inflammation. The direct colonization of intestinal ACE2 receptors through ingestion of the virus is potentially responsible for a range of gastrointestinal tract symptoms associated with COVID-19 [34,35]. Chronic obstructive pulmonary disease is often concomitant with chronic diseases of the gastrointestinal tract. Yet, there is a higher risk of allergic diseases of the airways and use of antibiotics and alteration in the composition of the gut microbiota [36]. Finally, a crosstalk pattern between Bacillus and Lactobacillus in the gut has recently been reported, revealing the extremely complicated interactions of multiple bacterial species in the gut microbiota [37].

The central role of the intestinal microbiota in the development of mucosal immunity is not surprising, as multiple interactions with the external environment take place in the gut and the intestinal epithelial barrier must tolerate the intestinal microbiota which constitutes the majority of the antigens presented to the resident immune cells [38]. Despite this condition, there is no strong activation of a local or systematic immune response. This condition occurs because tolerance is induced due to intestinal epithelial cells (ICE) which are in close contact with the intestinal microbiota and are constantly exposed to a large number of antigens [32,38]. In order to minimize the toxic potential of these antigens, ICEs adopt a number of strategies, such as reducing their TLRs, and modifying the antigenic fractions of the microbiota, to make them less immunogenic. The important role of the gut microbiota in the development of the systemic immune system has been assessed by studies conducted in the model of germ-free mice, i.e., without microbiota, born and kept in sterile conditions. These mice have various immune disorders, abnormal numbers of different types of immune cells, altered cytokines, as well as deficits in the local and systemic lymphoid structure [39].

The gut microbiota is a physical barrier to incoming pathogens through competitive exclusion, i.e., resistance to colonization, via mechanisms such as the occupation of attachment sites, the consumption of nutrients and the production of antimicrobial substances [40]. The interactions between antimicrobial peptides and microbiota are bidirectional. Gut bacteria secrete and consume a wide variety of neuromodulators and neurotransmitters, including serotonin, dopamine, gamma-aminobutyric acid, epinephrine and noradrenaline [41]. In this context, blood levels of serotonin, a metabolite of tryptophan independent of the kynurenine pathway, are lower in patients with severe COVID-19 than in healthy controls, suggesting that during SARS-CoV2 infection tryptophan is facilitated to take the kynurenine route [42].

A plethora of microbiota-derived compounds are produced as intermediates or final products of microbial metabolism and can influence biological functions both in the peripheral and the central nervous system (CNS) through nerve activation, cytokine production, neurotransmitters, and via systemic circulation [41,43]. The metabolites produced by the intestine not only modulate gastrointestinal immunity, but also affect distant organs, such as lung and brain.

A relevant response of the host’s immune system following microbial colonization of the gut is the production of immunoglobulin (Ig)A by gut-associated lymphoid tissues. IgA plays a vital role in mucosal homeostasis in the intestine and functions as the dominant antibody [43,44].

### 2.1. Inflammation and Dysbiosis of the Elderly Patient

The gut microbiota shows a great inter-individual variation and the human intestinal microbiome, i.e., the community that includes the genetic heritage and environmental interactions of all microorganisms, is very diverse and complex and continues to fluctuate during the various stages of life [43]. Furthermore, the intestinal microbiome is closely associated with various characteristics of integrity of the intestinal barrier, anti-inflammatory balance, immune and cardio-metabolic health, as well as the intestine–brain axis [41,44]. A loss of microbiota stability has been frequently observed in the elderly. The disruption of the intestinal barrier integrity as well as a condition of intestinal dysbiosis can further complicate the state of severity in SARS-CoV-2 [45,46]. In severe cases of SARS-CoV-2, elevated zonulin levels are a marker related to increased mortality. Measurement of LPS binding protein, a marker of inflammation, revealed a significant increase in more severe cases, supporting the association between severe COVID-19 and loss of intestinal barrier integrity and microbial translocation [47].

The excessive accumulation of senescent cells present in aging and age-related diseases can contribute to chronic silent inflammation and tissue and organic dysfunction. Furthermore, old-age problems could contribute to a greater predisposition to various infectious and associated diseases of the intestine causing alterations in the microbiota of the elderly [30,48,49].

Studies have shown a decrease in the diversity of gut microbiota composition in the elderly, and COVID-19 patients manifest faecal microbiome alterations, with an increase in opportunistic pathogens and a depletion of commensal beneficial microorganisms [14,15]. Older people are known to have a less diverse gut microbiota and a noticeable decrease in beneficial microorganisms such as *Bifidobacterium.* Since diet, drug intake and the composition of the gut microbiome undergo substantial changes during aging, the intestinal metabolic environment, and therefore the levels of microbial metabolites, are influenced by age. Gut microbial-derived metabolites play a key role in inflammatory signalling by interacting with host immune cells. Some bacterial species, such as *Faecalibacterium prausnitzii*, *Roseburia intestinalis*, and *Anaerostipes butyraticus* are able to digest complex carbohydrates by fermentation, generating short-chain fatty acids (SCFA), fatty acids with fewer than six carbon atoms, consisting mainly of acetate, propionate and butyrate [32,50,51]. The elderly has lower SCFA levels than young subjects. A decrease in SCFA production in the colon has been linked to lower fibre intake and antibiotic treatments in the elderly by regulating expression of pro-inflammatory cytokines including IL-6, IL-12 and TNF-α [52]. SCFAs are important for their ability to reduce intestinal inflammation, protect against pathogenic invasion and maintain barrier integrity primarily by activating G-protein-coupled receptors (GPCRs) or inducing their suppressive effects on histone deacetylase (HDAC), and by affecting gene expression. The genesis of the cytokine storm could take place in the gastrointestinal tract [41]. SCFAs maintain the physiology of the intestinal epithelium by regulating cell turnover and barrier functions. SCFAs constitute a key regulatory system for the activation, recruitment and differentiation of immune cells, including neutrophils, macrophages, dendritic cells (DCs) and T lymphocytes [41,50]. A reduced abundance of butyrate-producing species is found in COVID-19 patients [51] and it has been suggested that the use of butyrate-producing species in COVID-19 patients in order to maintain the integrity of epithelium at the level of tight junctions could likely help reduce invasion of SARS-CoV-2 [52].

The intestinal microbiota plays a crucial role in gastrointestinal physiology by providing, among other activities, the synthesis of endogenous vitamins, such as vitamin K and most of the components of the vitamin B complex [41]. In particular, vitamin B constitutes an important support for the correct activation of the immune response, and interestingly, it improves respiratory function, maintains the integrity of the endothelium and prevents hypercoagulability. A dysbiotic condition could lead to a vitamin B deficiency and could significantly impair immune function. Therefore, B vitamins could be a crucial aid in the treatment of SARS-CoV-2 [52].

Another interesting aspect of diet in the elderly is the consumption of proteins, and in particular a diet that includes an excessive amount of proteins can be responsible for an increase in the intestinal production of potentially deleterious bacterial metabolites. The requirement for a higher dose of protein in elderly subjects is suggested in order to compensate for the lower sensitivity to anabolic stimulus. However, the very amount of protein in the diet and additional amino acids can influence the onset and progression of inflammation [53,54,55]. This condition could also, in turn, affect epithelial repair since some bacterial metabolites inhibit respiration of colon epithelial cells, cell proliferation and/or the influence of barrier function.

Another element that characterizes the elderly subject is greater constipation leading to the use of laxatives, whose prolonged consumption has harmful effects on the entire intestinal ecosystem, loss of colon tone and a dangerous condition of habit [56,57]. There can be multiple causes of constipation, including eating disorders and dehydration, as well as pathological conditions, gastrointestinal pathologies, neurological or psychological causes [58].

Evidence suggests that age-related intestinal dysbiosis may contribute to unhealthy aging [59,60,61,62,63]. Since the gut microbiota communicates with the host through various biomolecules, pathways independent of the signalling of nutrients and epigenetic mechanisms, an alteration of these communication pathways related to age-related intestinal dysbiosis can heavily influence the health and life span of the host and trigger an innate immune response [45]. The circulation of bacterial compounds in the host is probably due to the breakdown of the intestinal epithelial barrier caused by the silent chronic inflammation state, and greater intestinal permeability has been suggested as a potential source of age-related inflammation [64,65,66].

The link between intestinal dysbiosis, chronic inflammation and fragility has been highlighted with intestinal permeability biomarkers [65]. These factors can lead to greater adherence and loss of various microbes and microbial derivatives and increase the host’s susceptibility to various local but also systemic disorders through the gut-brain axis, the gut- liver axis, the gut-lung axis [67]. Therefore, in this context, microbial metabolites play an important role in human longevity. However, it is not clear whether the condition of intestinal dysbiosis is a cause or rather a consequence of aging and associated inflammatory disorders. The composition of the gut microbiota is related to circulating cytokine levels and health indicators in the elderly [12]. If the gut microbiota is an age-associated inflammation factor, this would mean that age-related changes in the gut microbiota represent a form of microbial dysbiosis.

A physiological translocation of microbial products is present throughout life; however, with aging, this microbial translocation increases and favours dysbiosis conditions. This feed-forward process increases over the years. In conditions of alteration of the epithelial barrier, the COVID-19 virus finds a fertile ground. Age-associated inflammation is a strong risk factor for mortality in the elderly. Patients with higher levels of inflammatory markers are more likely to be hospitalized, and have higher mortality rates, are fragile, are less independent and are more likely to experience late disease [64,67]. Finally, inflammation in the elderly increases the susceptibility to pneumococcal infection, and is associated with a rise in disease severity and reduced survival [68].

Current research confirms that the intestinal microbiota is significantly altered in SARS-CoV-2 infection, highlighting the crucial role of microbiota in modulating the human response to SARS-CoV-2 infection [25,28]. Interestingly, the alterations are characterized by an opportunistic growth of pathogens while, at the same time, there is a dramatic decrease in beneficial commensal microorganisms [25].

Studies have reported the intimate relationship between infection and gut microbiota dysbiosis and have shown that infection is associated not only with gut bacteria but also with resident viruses. A study reported that treatment with *Lactobacillus brevis* OW38 to aged mice reduced the lipopolysaccharide (LPS) level in colon fluid and blood. Administration of *Lactobacillus brevis* OW38 reduced the ratio of Firmicutes or Proteobacteria to Bacteroidetes. In addition, this lactic acid bacterium was able to inhibit the expression of inflammatory markers, such as myeloperoxidase, TNF, and IL-1β, and inhibited NF-κB activation [69].

### 2.2. Microbiota and Lung

**Figure 1:**
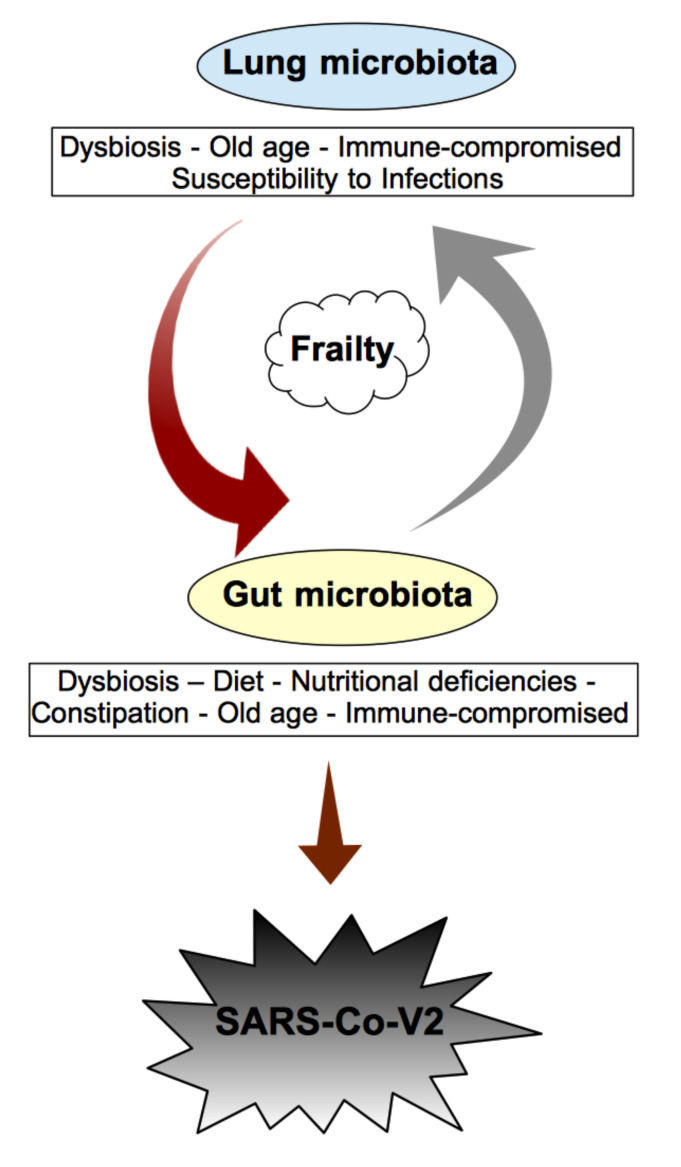
Schematic representation of the bidirectional link between lung and gut. The gut microbiota influences lung health through a cross dialogue between the gut microbiota and the lungs, the “gut–lung axis”. The functional state of the elderly, dysbiotic condition, immune compromise, nutritional deficiencies constitute as a whole, a condition of extreme vulnerability.

The lung microbiota is less relevant in quantity than the gastrointestinal microbiota; however, it is originally colonized by the oropharynx and by microaspirations of the gastrointestinal tract. The predominant bacterial phyla both in the lungs and gut are the same, Firmicutes and Bacteroidetes [70]. The fungal component is also prominent, which is known to communicate with bacteria. The gut and lung microbiota are in parallel throughout life, although dietary changes affect not only the gut microbiota but also the lung microbiome [71,72]. Bidirectional crosstalk has been demonstrated in animal experiments [73]. Members of the gut microbiome induce immune tolerance and block the colonization of pathogens through the activation of the immune system and the direct and indirect actions of the microbiota. When the immune system “learns” to recognize the enemy from the microbiome, the effect can also occur in a distant organ [74]. Various studies have shown that lung infections are associated and mutually influenced with a change in the gut microbiota [75] (Figure 1).

COVID-19 represents a further aggravation of the inflammatory problem as age-associated inflammation causes macrophage dysfunction and tissue damage. An increase in circulating bacterial toxins implies a reduction in the gene expression of tight junctions and lethal lung damage [71]. Aging is characterized by a particular condition, the so-called “chronic age-related inflammation”. This condition is genetically preordained and is a chronic inflammatory process with a shift in the profile of proinflammatory cytokines at the level of the various districts with the presence of greater amounts of histamine, IL-1 and TNF cytokines and chemotactic factors. In the elderly, it is a consequence of the long-term antigenic load with a continuous involvement of the immune system. The functional degradation of the immune system that occurs with aging is linked to changes in immune-competitive cells and other cells. The changes affect the size of cells, but also their functions and population size. In the elderly, chemotaxis, phagocytosis and antigen presentation worsen in a context of high level of proinflammatory cytokines. Excessive cytokine production leads to chronic overstimulation of the immune system [72].

Like the gastrointestinal tract, lungs are at the forefront of immunity as they are constantly attacked by a wide variety of external environmental stimuli. The microbiome of the lungs plays a crucial role in shaping and harmonizing lung immunity. As in the intestine, the lung microbiota has the task of strengthening innate and adaptive immunity, releasing factors that support respiratory functions and defend the lungs from pathogens [75]. Intestinal dysbiosis has been implicated in various lung diseases, such as asthma and cystic fibrosis. Diet alters the microbiome. So, an altered lung microbiome predicts disease progression in interstitial lung disease [76,77].

Studies have reported the role of fibre-rich diets in modulating innate immunity, supported by a reduction in inflammatory marker levels [77]. A diet rich in fibre influences and modifies not only the intestinal microbiota, but also the lung microbiota, supporting the role of nutrition on lung immunity [77,78,79].

The depletion of some species of the intestinal microbiota due to the intake of antibiotics influences lung diseases and allergic inflammation [80,81]. In mice it has been observed that influenza virus infection in the respiratory tract increases Enterobacteriaceae and reduces Lactobacilli and Lactococci in the gut microbiota [82]. Dysbiosis in the lung microbiota after LPS administration is accompanied by disorders of the intestinal microbiota due to the movement of bacteria from their lung into the bloodstream [83].

Lactobacilli and Bifidobacteria are beneficial probiotics that exert a trophic effect on the intestinal mucosa. They can promote host defence against infections and reduce hypersensitivity reactions to commensal bacteria and antigens. Specific selected probiotic strains are capable of modulating the expression of proinflammatory molecules and anti-inflammatory properties. The anti-inflammatory and preventive abilities of specific probiotic mixtures have been described [84,85,86]. Interestingly, M1 macrophages, which produce proinflammatory cytokines, such as IL-6, and M2 macrophages which produce anti-inflammatory cytokines, such as IL-10, can be modulated by specific probiotic treatments [85]. Finally, already various studies have reported that respiratory viral infections can affect the gut microbiome condition, including pulmonary influenza virus and respiratory syncytial infections [73,87].

## 3. Focus on Mast Cells, SARS-CoV-2 and Microbiota

In the context of the wide variety of cells involved in SARS-CoV-2, two types of cells that are at the forefront of the pathogenesis are mast cells (MCs) and platelets, and a control over them could represent biomarkers and targets at the same time for an interesting therapeutic strategy.

MCs are innate immunity cells present in mucous membranes and connective tissue, strategically located at the interface with the external environment such as the skin, lungs and intestines, where they act as gatekeepers for attack of pathogens [70,88], and they play, themselves, pathogenic roles in many inflammatory responses. MCs organize the inflammatory response and are crucial early participants in responses to viral infection. Once activated, in a very short time MCs release mediators classified as dependent or independent of degranulation. These molecules contribute to inflammation and changes at the site of infection. Mast cells also can be activated by a variety of both bacterial and viral products, and, consequently, to release a very wide spectrum of proinflammatory and immuno-regulatory molecules. In addition, many studies have analysed the ability of MCs to contract common viruses and release molecules such as histamine and leukotrienes [89,90]. MCs are resistant to productive infection with respiratory syncytial virus but have a protective response that includes the production of cytokines and chemokines that promote the recruitment of antiviral effector cells [89]. MCs can be activated directly by active viral infection or by contact with viral particles. Activation of MCs leads to the production of a variety of mediators, including large amounts of interferons (IFNs) by human virus-infected cells. In addition to initiating an antiviral state in neighbouring cells, a storm of chemokines and cytokines promote the local recruitment of effector cells. IFN also acts in an autocrine manner to further promote the production of MCs. The molecules released by MCs also act by improving lymph nodes hypertrophy. Furthermore, the involvement of local dendritic cells promotes the development of a subsequent acquired immune response [91]. Mast cells can influence T cell proliferation and cytokine production [92]. In addition, MCs produce proteases that are increased in COVID-19 sera and lung districts [93]. Mast cells contain the serine protease ACE2 [94].

MCs play a leading role in many pathophysiological conditions, in which there is a condition of chronic silent inflammation. IL-1β, IL-6 and IL-8 are typical of silent chronic inflammation and MCs are both producers and effectors of these cytokines. Furthermore, MCs are involved in inflammatory responses and psychological stress [70]. MCs are about 2–3% of the immune cellular pool of the lamina propria, and in the muscular and serous layers (3000–25,000 MCs/mm^3^) [10]. Variations in the number of MCs are observed in the elderly, as with aging there are changes in connective and mucous tissues as well as other changes closely related to CNS disorders, including depression and anxiety [95]. An increase in the number of MCs during aging has also been observed in human organs and organs of other mammals and vertebrate animals.

MCs play a crucial role in host–microbiota communication, as they can help influence microbiota status and host conditions by modifying their activation [10,96]. MCs can contribute to the maintenance of intestinal homeostasis and their activation is linked to a variety of factors, motor abnormalities and dysfunctions of the intestinal epithelial barrier [10,26]. MCs establish functional signalling pathways with the nervous system and nerves in the gut. Their activation induces sensitization of the nerves, and these, in turn, can condition the release of mediators from MCs. This crosstalk is critical in the generation of symptoms or in the pathogenesis of inflammatory disorders [97,98].

MC responses to virus and other pathogens provide excellent tools for modifying local immune responses and could represent an attractive target for COVID-19 treatment, vaccination, and other immunotherapeutic uses. As is known, MCs can be activated by PAMPS through TLRs. Interestingly, MCs have been shown to express the renin–angiotensin system, the angiotensin 2 converting enzyme ectoprotease required for binding of SARS-CoV-2 and serine proteases [70,99]. This could lead to the secretion of proinflammatory mediators in a targeted and selective manner, without release of histamine or tryptase, as has already been described for the release of IL-6 in response to IL-1β from human MC cultures [70]. 

MCs could be a potential target to control SARS–CoV-2, for example employing known MC stabilizing agents [96]. Interestingly, some specific probiotic strains are able to stabilize MCs, especially *L. rhamnosus* GG [100]. Oral administration with *L. rhamnosus* JB-1 induces inhibition of peritoneal MC degranulation [101].

Relationships established between gut microbiota composition, cytokine storm, and MC activation in SARS-CoV-2 patients suggest that the gut microbiome is extremely involved in the severity of the pathology. Finally, an intestinal dysbiosis condition could then contribute to the condition of feeding those persistent symptoms that characterize the outcomes of SARS-CoV-2.

## 4. Focus on Platelets, Microbiota and SARS-CoV-2

Platelets play an important role in a variety of regulatory and degenerative processes [102]. Platelets participate in inflammation by producing a variety of pro-inflammatory molecules [103]. COVID-19 is associated with increased production of large immature platelets, as megakaryocytes respond to increased platelet consumption. Circulating IL-1β, IL-6 and IL-8 are not regulated in chronic systemic and silent inflammation and also have receptors on platelets [103]. Platelet hyperactivation is observed in aging. And it is unclear whether such hyperactivity is the cause or effect of various other vascular disorders in the elderly [104]. Platelets can interact with viruses through a variety of receptors, including TLRs. The role of platelets in haemostasis is well known, and hypercoagulability is an important sign of inflammation. In particular, IL-1β, IL-6 and IL-8 are critically involved in the formation of abnormal clots, erythrocyte pathology and platelet hyperactivation. The most relevant changes were detected when all three cytokines caused platelet hyperactivation and spread with vessel damage and thrombogenic effects [105]. Interestingly, a metabolite of the gut microbiota, called phenylacetylglutamine, was recently identified as being able to enhance platelet activation-related phenotypes, thus favouring platelet hyperactivation. This metabolite could therefore increase the thrombotic capacity and increase the risk of cardiovascular complications [106]. In this context, a targeted control of the microbiota could counter the development of such cardiovascular diseases.

**Figure 2:**
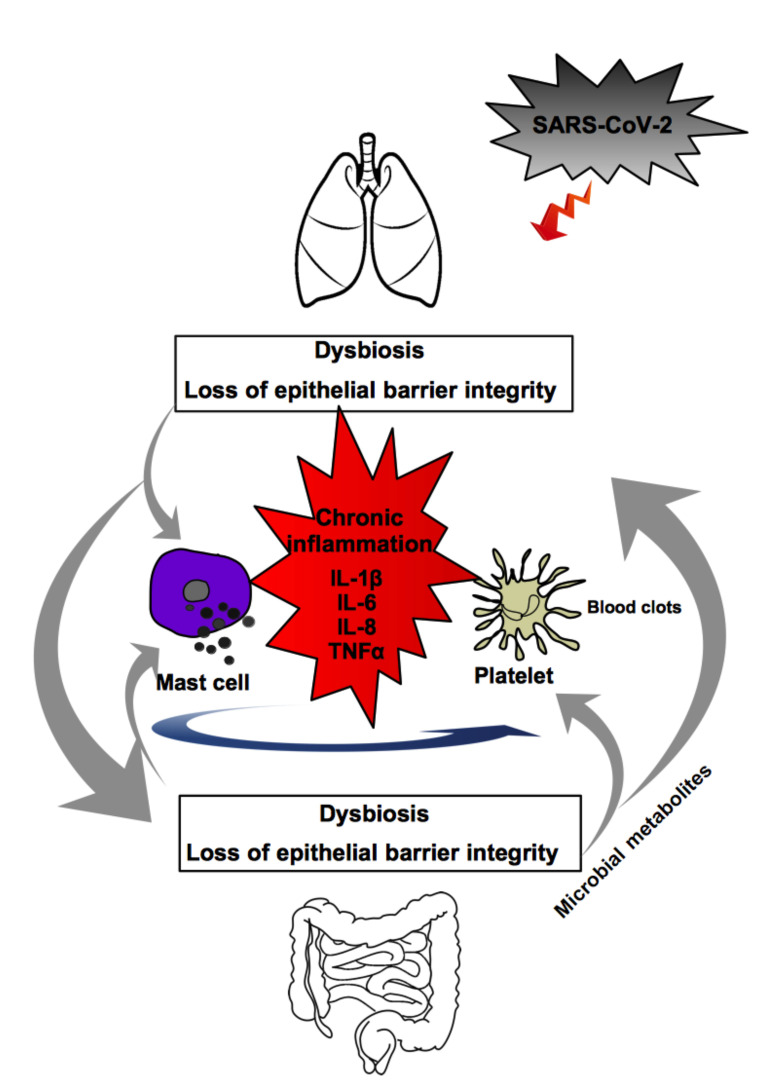
Drawing illustrating the crosstalk between the various players discussed in the paper. In the elderly subject there is a state of fragility of the lung and intestinal microbiota, with loss of the integrity of the epithelial barriers, and chronic silent inflammatory state. Activated mast cells produce a wide variety of cytokines, chemokines and other inflammatory mediators that extensively influence and condition the gut and lung microbiota composition in a vicious cycle; these mediators also affect platelets which, in turn, are affected by microbial metabolites.

Numerous cases of thrombocytopenia have been detected in patients with COVID-19 and three mechanisms have been hypothesized to explain the phenomenon: (i) the virus can directly infect bone marrow cells and inhibit platelet synthesis. The cytokine storm destroys progenitor cells and leads to reduced platelet production; (ii) the immune system destroys platelets; (iii) platelets aggregate in the lungs, resulting in the consumption of micro-thrombi and platelets [107]. The production of cytokines induced by a dysbiotic microbiota, the activating effect that inflammatory stimuli exert on platelets, MCs and astrocytes, allow the release of further pro-inflammatory molecules, involving an amplification of the harmful effect, micro-thrombi and, considering the location of MCs near the nerves, even possible neurological and brain damage, up to psychopathological conditions, anxiety and depressive syndromes [108]. Finally, it has recently been shown that the lung contributes to platelet biogenesis [109]. Therefore, platelets play a crucial role in the pathogenesis of SARS-CoV-2, as they release various types of molecules through the different stages of the disease. Platelets may have important potential to contribute to the thrombus-inflammation that occurs in SARS-CoV-2, and an inhibition of pathways related to platelet activation could significantly improve outcomes during COVID-19. It has been shown that *L. plantarum*, *L. rhamnosus* and *L. acidophilus* can control any platelet activation [110] (Figure 2).

## 5. Interventions with Antiviral Bacteria

Gut microbiota control could have distal protective effects on antiviral responses. There is evidence of the role of inflammasome activation in immune defence against influenza virus infection [111,112].

The commensal respiratory bacteria, *Corynebacterium pseudodiphtheriticum* modulates the TLR3 antiviral response against respiratory syncytial virus, enhancing the production of TNFα, IL-6, IFNγ and IFNβ by increasing the T cell subpopulations that produce these cytokines [113]. The protective role of commensal bacteria, mainly probiotics, is now well established. Specific probiotics such as lactic bacteria, are actually considered friendly bacteria, and secrete antiviral substances during their growth [113,114]. A dialogue is established between the intestinal microbiota and that of the airways through the intestine-lung axis and it could explain how gut bacteria are able to enhance antiviral immunity as gut microbial metabolites could stimulate immune cells that, in turn, could move distally and mediate an antiviral response [112,114].

*Lactobacillus paracasei* and *L. plantarum* were able to reduce the inflammatory response in the lungs by increasing IL-10, and thus controlling the antiviral response [115,116]. Studies have shown the action of *L. gasseri* in various viral infections, including respiratory infections. Recent reports indicate *L. gasseri* SBT2055 is a promising probiotic useful for the prevention of human respiratory syncytial virus [117]. Finally, other nutritional interventions for coronavirus infection control have also been suggested, such as reducing the consumption of purine food sources [118], as it has been suggested that coronaviruses use purine nucleotides to promote synthesis of RNA [119].

Some possible mechanisms of antiviral activity mediated by bacteria could be the following: (i) bacteria could prevent the adsorption and cellular internalization of the virus by trapping it; (ii) bacteria could establish a link with cells to organize antiviral protection; (iii) microbial metabolites could have a direct antiviral effect.

Interestingly, some probiotic strains show antiviral activity against some coronaviruses [120]. Selective probiotic strains are able to control the levels of type I interferons, increase the number and activity of antigen presenting cells, NK cells, T lymphocytes, specific antibody levels in the lungs [119]. Specific probiotic strains are also capable of modifying the dynamic balance between proinflammatory and immunoregulatory cytokines that allow viral clearance while minimizing lung damage mediated by the immune response. *Bifidobacterium longum SP 07/3, L. gasseri* PA 16/8, and *Bifidobacterium bifidum* MF 20/5 contribute to reducing the duration of common cold episodes but also days with fever [121]. This could be especially important in preventing COVID-19 complications. A randomized clinical trial with *L. plantarum* DR7 showed suppression of plasma proinflammatory cytokines, such as IFN-γ, TNF-α in adult patients and potentiation of anti-inflammatory cytokines in young adults, along with a reduction in oxidative stress levels [122].

Strategies could be developed to alter the gut microbiome in order to manage the gastrointestinal effects of the virus in elderly COVID-19 patients and also to control the lung microbiota.

## 6. Conclusions

Over a century ago, in his book, Metchnikoff [123] suggested that the manipulation of the gut microbiota could prolong life. A dysregulated immune response may cause lung immunopathology. Strategies to combat multifaceted COVID-19 could be to reduce age-associated inflammation, delay the onset of disease inflammation and prolong life. The functional state of the elderly, their dysbiotic condition, immune-compromised with nutritional deficiencies constitutes as a whole, a condition of extreme vulnerability.

Intestinal microbiota dysbiosis is strongly associated with the pathogenesis of several metabolic and inflammatory diseases and the control of the intestinal microbiota could represent a certain challenge to COVID-19, now and even later, in the consequences that SARS-CoV-2 will bring on the general population.

Changes in the microbial population of the elderly and the associated decline in intestinal tissue function can fuel a chronic state of inflammation, resulting in a vicious cycle that further affects host–microbiome interactions and amplifies the frailty of the elderly. On the other hand, chronic immune stimulation as a consequence of silent systemic inflammation and changes in the metabolome and microbial stimuli contribute to immune senescence.
